# Comparison of 2D 4K vs. 3D HD laparoscopic imaging systems using a pelvitrainer model: a randomized controlled study

**DOI:** 10.1007/s13304-021-01195-0

**Published:** 2021-10-26

**Authors:** Tibor A. Zwimpfer, Claudine Wismer, Bernhard Fellmann-Fischer, James Geiger, Andreas Schötzau, Viola Heinzelmann-Schwarz

**Affiliations:** 1 Department of Obstetrics and Gynaecology, University Hospital Basel, Spitalstrasse 21, 4056 Basel, Switzerland; 2 Department of Biomedicine, University and University Hospital Basel, Basel, Switzerland; 3 Medical Faculty, University of Basel, Basel, Switzerland

**Keywords:** Laparoscopy, 2D 4K vision system, 3D HD vision system, Pelvitrainer, Standardized tasks

## Abstract

Laparoscopic surgery provides well-known benefits, but it has technological limitations. Depth perception is particularly crucial, with three-dimensional (3D) imaging being superior to two-dimensional (2D) HD imaging. However, with the introduction of 4K resolution monitors, 2D rendering is capable of providing higher-quality visuals. Therefore, this study aimed to compare 3D HD and 2D 4K imaging using a pelvitrainer model. Eight experts and 32 medical students were performing the same four standardized tasks using 2D 4K and 3D HD imaging systems. Task completion time and the number of errors made were recorded. The Wilcoxon test and mixed-effects models were used to analyze the results. Students were significantly faster in all four tasks when using the 3D HD perspective. The median difference ranged from 18 s in task 3 (*P* < 0.003) up to 177.5 s in task 4 (*P* < 0.001). With the exception of task 4, students demonstrated significantly fewer errors in all tasks involving 3D HD imaging. The experts’ results confirmed these findings, as they were also faster in all four tasks using 3D HD, which was significant for task 1 (*P* < 0.001) and task 4 (*P* < 0.006). The expert group also achieved better movement accuracy using the 3D HD system, with fewer mistakes made in all four tasks, which was significant in task 4 (*P* < 0.001). Participants in both groups achieved better results with the 3D HD imaging system than with the 2D 4K system. The 3D HD image system should be used when available. Trial registration: this trial is registered at research registry under the identifier researchregistry6852.

## Introduction

Laparoscopy has clear benefits in comparison to open surgery. Specifically, it enables reduced blood loss, a significant decline in the postoperative infection rate, shorter hospitalizations, and faster recovery times [1–3]. However, it has disadvantages that include technical limitations, limited degrees of freedom, unnatural ergonomics, and limitations in current instrument design and visualization [4].

Robotic surgery has eliminated some of these disadvantages. Superior 3D HD vision has improved the well-being of surgeons through better ergonomics, and improved freedom has led to more complex procedures being performed [5–7]. However, high acquisition and maintenance costs, with no clear benefits in terms of inpatient hospital stays or operation times, mean the advantages of robotic-assisted laparoscopy do not completely outweigh the need for the conventional procedure [8, 9].

Recently, various approaches have been used to overcome technical limitations including improved ergonomics [10–12] and vision [13–16]. In comparison to the 2D HD system, one advantage of the 3D HD image system has been to increase surgeon self-confidence during operations due to the subjective impression of safety and efficiency. Furthermore, the 3D HD image system significantly reduces operation time and blood loss, improves visibility, and shortens hospital stays [17]. However, while the 3D imaging system leads to faster results and better depth perception, the system is not commonly used, as there is a need to educate nurses in the technology and modernize operating rooms, both of which entail higher costs [18, 19].

Currently, the new 4K imaging system is becoming available. This system promises to improve vision through ultra-high definition, a wider range of colors, and augmented visualization [20]. 4K has a resolution of approximately 4000 × 2000 pixels which is four times higher than HD resolution and provides four times more information than conventional HD imaging systems [21]. Improved visibility enables surgeons to look at tissue structures at close proximity and assess it more accurate [20]. Additionally, Wider Color Gamut enables rich color reproducibility and provides a more realistic image with better visualization of blood vessels and lesions [20].

Both novices and experienced surgeons have been shown to obtain better results regarding operation time and errors made, and in reducing repetitions when using the 2D 4K system rather than the 2D HD one [22]. A study by Abdelrahman et al*.* [22] found that students using the 2D 4K resolution showed a significant reduction in errors, but the needed time was balanced in comparison with 3D HD imaging. However, Kanaji et al. [23] found that subjects with no prior experience demonstrated better laparoscopic performance using 3D HD monitors rather than the 2D 4K ones. Currently, the 2D 4K technology could become standard in the near future, and expert surgeons may have advantages in performing tasks within narrow spaces using 2D 4K imaging [24]. Therefore, in this study, we used four standardized tasks in a pelvitrainer model to compare expert and student performances when using the latest 2D 4K system in comparison to the 3D HD vision system.

## Materials and methods

### Study population

There were 40 study participants, and completed data were obtained from all subjects. Eight participants were specialists with a surgical or gynecological background and 32 were medical students. For the purpose of this study, an expert was defined as someone who had conducted more than 30 laparoscopic operations per year for a minimum of 5 years and had a gynecological or surgical background. A student was defined as someone in training with no experience in laparoscopy, and recruited from the University Basel. All test subjects were right-handed.

The Lang-Stereotest II was used to ensure that none of the participants had problems with stereoscopic vision [25]. All of the participants gave their written consent to participate in the study. The anonymization of personal data was guaranteed. The project was not defined as a research project according to the Human Research Act Article 2; therefore, an IRB approval was not required.

### Study design

**Fig. 1:**
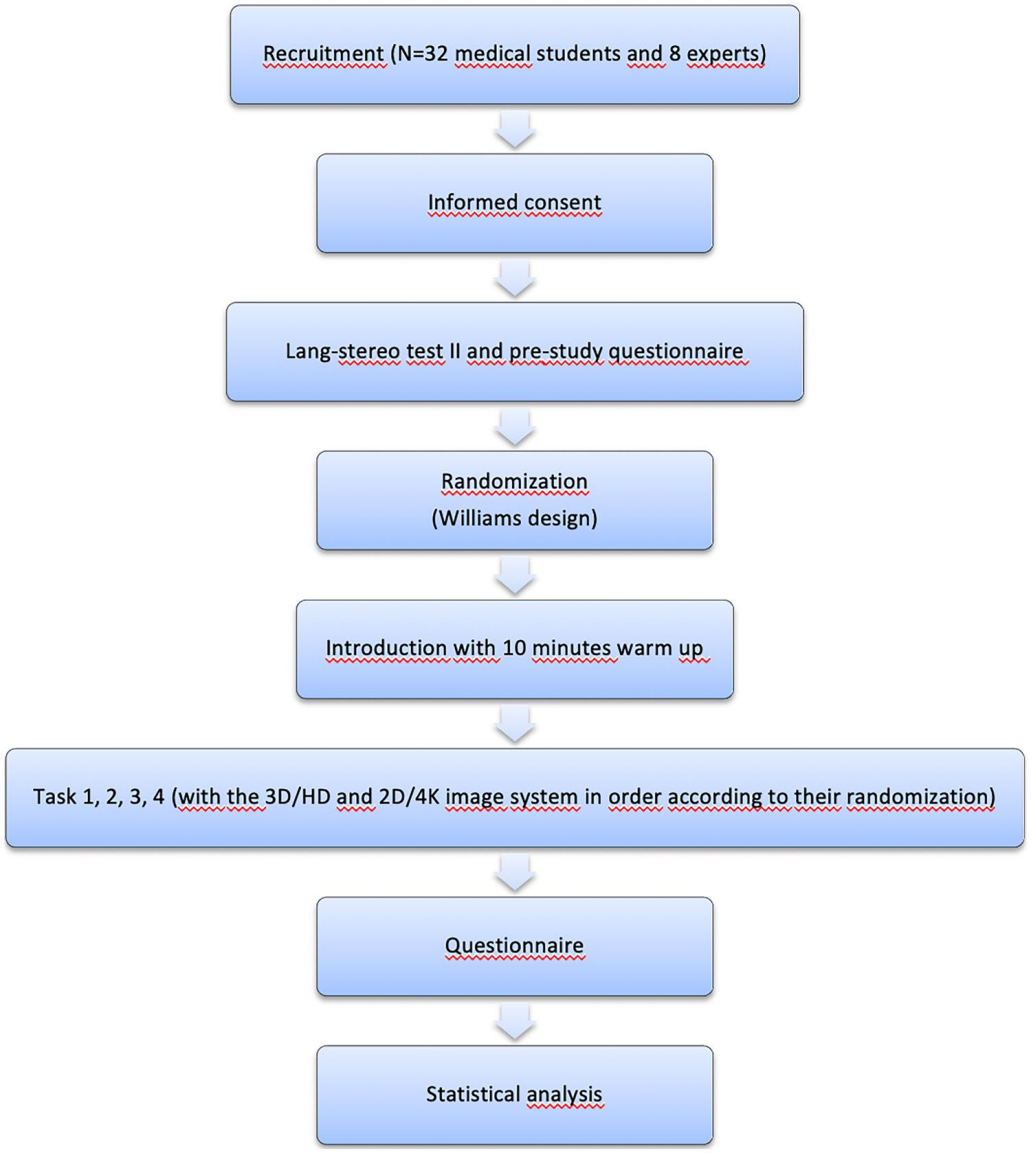
Flow diagram illustrating study design and process

Each participant performed 4 standardized tests with the 3D HD and the 2D 4K systems. Using the Williams design, subjects were randomly assigned to perform the tasks either first using the 3D HD system and then the 2D 4K or vice versa [26]. We used the 3D HD and 2D 4K systems by Karl Storz (Karl Storz SE & Co., Tuttlingen, Germany). The time taken to complete each task and the errors made during the tasks were recorded during each subject’s performance. Once the participants had completed all of the tasks, they were given a questionnaire asking about their experience with both systems (Fig. 1).

### Instructions

Before each task, participants watched an introductory video that including detailed instructions. The participants had a maximum of ten minutes to familiarize themselves with the instruments and the pelvitrainer. If the participants had any additional questions, they were given the opportunity to ask them prior to beginning the tasks.

### Instrument set-up

All exercises were carried out on a pelvitrainer constructed such that it correlated with the abdomen and pelvis of a human. An endoscopy tower with a 32″ 4K/3D monitor (3840 × 2160 screen resolution) was also put in place (Karl Storz SE & Co., Tuttlingen, Germany) which is compatible for 3D HD and 3D 4K. For the 2D 4K system, a Hopkins II, 10 mm, 30° telescope was used, while a TIPCAM1 S 3D, LAP Optic, 10.3 mm, 30° telescope was used for the 3D system, both used the Image 1 S™ 4U camera system (Karl Storz SE & Co., Tuttlingen, Germany). For the 3D system, 3D glasses were provided by Karl Storz SE & Co., Tuttlingen, Germany. The participants were positioned exactly one and a half meter in front of the monitor to guarantee the effect of both, the 4K and 3D imaging system.

### Tasks

Tasks were designed to imitate real surgical scenarios and test different laparoscopic skills including precision, speed, and dexterity. To measure the amount of time it took for each task to be completed, areas were marked to define the initial position of the laparoscopic instruments. Each task started and ended at this position. Errors were recorded and measured using an automatic fault counter for the objective evaluations of tasks 1, 2, and 3. For these purposes, the laparoscopic clamps, as well as the area that was off-limits during the exercise, were connected to the counter. Errors were manually counted for a subjective assessment in tasks 2 and 4. A mobile phone with a start/stop feature was used to record the time elapsed at the end of every task and was used to measure the time required for each task to be completed. The four tasks followed a procedure that mimicked that of Zwimpfer et al. [14].

#### Task 1: mountain relief (orientation using 2D 4K and 3D views)

**Fig. 2:**
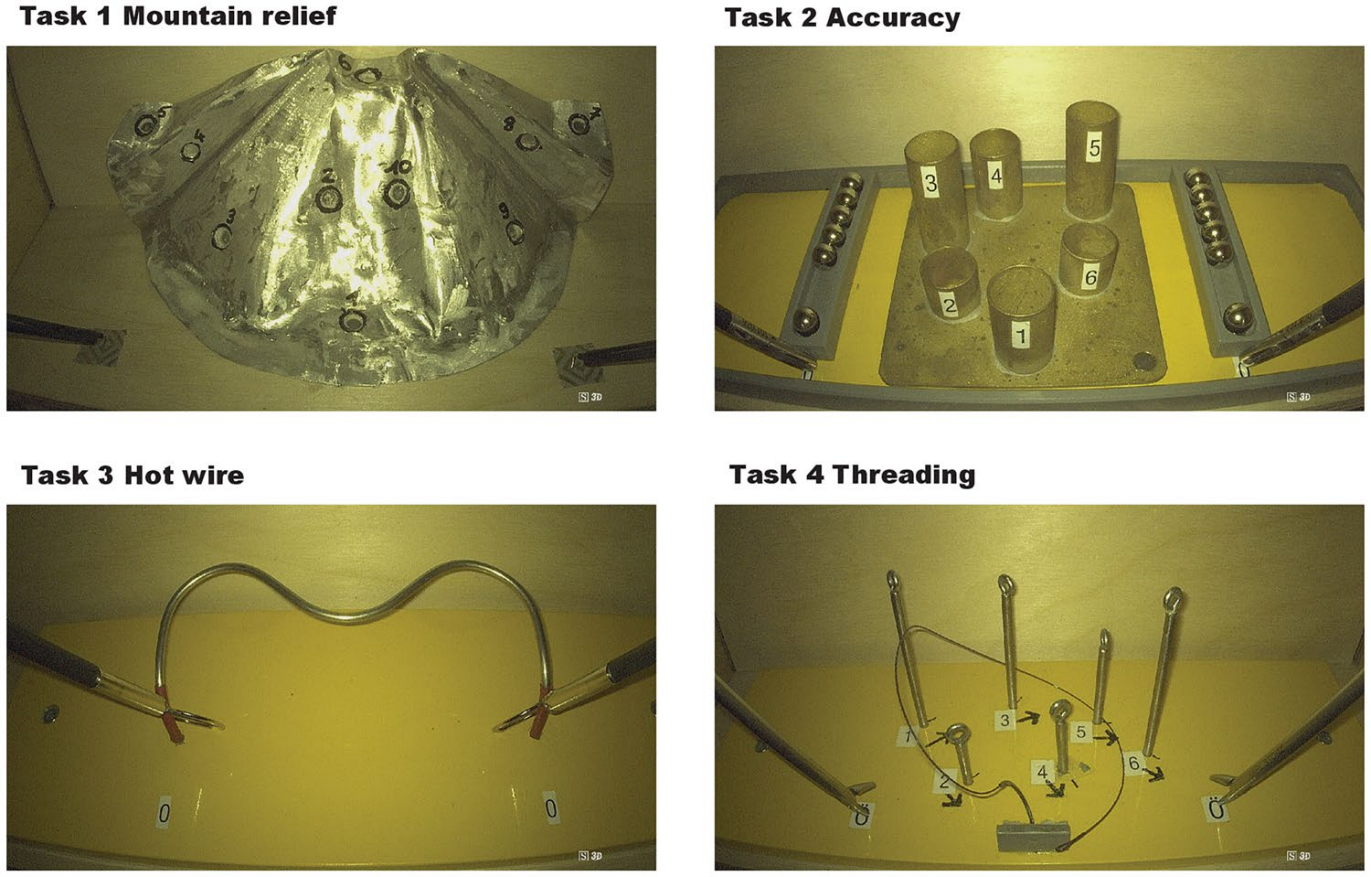
Illustration of the start position of tasks 1 through 4 with the 3D HD image system

In this task, ten numbered notches were positioned on a circle. The goal was to touch only the inner side of a notch using a monopolar coagulation electrode. When a notch was successfully contacted, it produced a sound. The task began with the right-hand instrument making contact with notch number one and continued up to notch ten. Once completed, the participants repeated the task with their left hand. Contacting the mountain in the wrong area or missing a notch was recorded as an error. The participants were not informed of their errors during the task (Fig. 2).

#### Task 2: accuracy (measuring precision when targeting defined points of the movements of an object from location A–B)

Six empty tubes were placed in a circle on a base plate with two ball containers holding six ferromagnetic balls located on both the right and left sides of the tubes. The goal was to take the balls from the containers and place them inside the tubes without touching the tubes. After the start signal, the participant took the first ball from a container with the right-hand instrument and loaded tube number 1. They continued with the other tubes in a clockwise direction. Once the right-hand side was completed, the participant performed the same task with the left-hand instrument. If a ball was dropped, it was abandoned and the participant continued on with the next one. Touching the tubes or dropping a ball in the wrong tube or on the ground was recorded both manually and automatically as an error. For this task, laparoscopic Kelly forceps were used (Fig. 2).

#### Task 3: hot wire (measuring accuracy, coordination, and time for non-linear and continuous movements)

A bent wire, insulated on both ends to rule out starting errors, was attached to a base plate. Two rings with a detached bracket were threaded onto the wire. The purpose of the task was to move the ring along the wire without making any contact. The participant started with the right-hand instrument and then switched to the left-hand one. Touching the wire with the ring was recorded as an error. There was a red insulated area at the beginning and end of the wire where subjects could position the rings without any errors being recorded (Fig. 2).

#### Task 4: threading (measuring handling and coordination using a needle holder and needle)

Six eyelets were positioned on a base plate, and they were numbered and assigned with a directional mark. The goal of this exercise was to thread the V-Lock-Needle through the eyelets. After the first eyelet, the needle needed to be threaded through the loop of the v-lock-thread. If an eyelet was threaded in the wrong direction or order, or if a knot was produced, it was recorded as an error. A maximum of twenty minutes was given to complete the exercise. For this task, straight-tipped needle holders from Karl Storz were used (Fig. 2).

### Questionnaires

Before and after completing the tasks, participants answered questionnaires. The questionnaire given before the tasks collected data concerning general information about the participants including sex, age, whether or not they played video games, the types of sports they practiced, and questions ensuring that the participants fulfilled the study requirements. For experts, the questionnaires also asked about experience level (in years) and the subject’s medical specialty. For students, they were asked which medical specialty they wished to pursue. After each task, the participants answered another questionnaire concerning how they felt both mentally and physically, and about the difficulty level of the task. After all of the exercises were completed, participants answered questions about their experience using the 3D HD and 2D 4K systems.

### Statistical analysis

For manually measured errors, the non-parametric Wilcoxon test was used to analyze the data. For the other tests, we used the parametric mixed-effects model and specified the mean ratio, which approximately corresponded to the median. Overall *P* values reported here correspond to the ANOVA *t* test for the means, the Kruskal-Wallis test for the medians, and the Chi-squared or Fisher’s exact test when the expected frequencies were less than 5. A *P* value < 0.05 was considered significant. All analyses were performed using the statistical software R (R version 4.0.0).

## Results

### Task 1: mountain relief

Overall, the participants were significantly faster and made fewer mistakes (*P* < 0.051) with the 3D HD system. The students had a median completion time of 228.5 s with the 2D 4K system and 132.5 s with the 3D HD system (*P* < 0.001). Additionally, the students made significantly fewer mistakes with the 3D HD system (*P* < 0.051). The experts were faster and made fewer mistakes compared to the students with both systems. They completed the tasks in a median of 156 s and 11.5 mistakes with the 2D 4K system versus 86 s and 7 mistakes with the 3D HD system (*P* < 0.001 and *P* < 0.330, respectively) (Tables 1, 2, and 3).

**Table 1:** Manually measured mistakes made in each task by experience group

Experience level	Contrast	Task	Median of the differences	95% confidence interval		*P* value
				Lower	Upper	
Students	3D/HD–2D/4K	1	0	0	0	NA
Students	3D/HD–2D/4K	2	2	2	3	< 0.001
Students	3D/HD–2D/4K	3	0	0	0	NA
Students	3D/HD–2D/4K	4	0	0	0	0.0593
Experts	3D/HD–2D/4K	1	0	0	0	NA
Experts	3D/HD–2D/4K	2	1	0	3	0.265
Experts	3D/HD–2D/4K	3	0	0	0	NA
Experts	3D/HD–2D/4K	4	0	− 0.5	0	0.3458

*NA* not applicable

The *P* value was calculated using the non-parametric Wilcoxon test. *P* < 0.05 was considered significant. For task 1 and 3, the manually measured mistakes were not collected

**Table 2:** Automatically measured mistakes made in each task by experience group

Status	Contrast	Task	Geometric mean ratio	95% confidence interval		*P* value
				Lower	Upper	
Students	3D/HD–2D/4K	1	0.7452	0.5546	1.001	0.051
Students	3D/HD–2D/4K	2	0.8954	0.6664	1.203	0.4618
Students	3D/HD–2D/4K	3	0.3128	0.2328	0.4203	< 0.001
Students	3D/HD–2D/4K	4	1	0.7443	1.344	1
Experts	3D/HD–2D/4K	1	0.7464	0.4107	1.357	0.3301
Experts	3D/HD–2D/4K	2	1.167	0.6418	2.12	0.6067
Experts	3D/HD–2D/4K	3	0.2403	0.1322	0.4368	< 0.001
Experts	3D/HD–2D/4K	4	1	0.5502	1.817	1

The *P* value was calculated using the parametric mixed-effects model. The mean ratio approximately corresponded to the median. A *P* value < 0.05 was considered significant. For task 4 the automatically measured mistakes were not collected

**Table 3:** Time measured in seconds made in each task by experience group

Status	Contrast	Task	Geometric mean ratio	95% confidence interval		*P* value
				Lower	Upper	
Students	3D/HD–2D/4K	1	0.5622	0.4859	0.6504	< 0.001
Students	3D/HD–2D/4K	2	0.8027	0.6939	0.9287	0.0033
Students	3D/HD–2D/4K	3	0.802	0.6932	0.9278	0.0032
Students	3D/HD–2D/4K	4	0.6053	0.5232	0.7002	< 0.001
Experts	3D/HD–2D/4K	1	0.6144	0.485	0.7783	< 0.001
Experts	3D/HD–2D/4K	2	0.9091	0.7177	1.152	0.4218
Experts	3D/HD–2D/4K	3	0.9446	0.7457	1.197	0.6303
Experts	3D/HD–2D/4K	4	0.7146	0.5641	0.9052	0.0063

The *P* value was calculated using the parametric mixed-effects model. The mean ratio approximately corresponded to the median. A *P* value < 0.05 was considered significant

### Task 2: accuracy

All experience groups spent less time and made fewer mistakes when using the 3D HD system. However, there was no significant difference in the mistakes made between the two imaging systems. The students were significantly faster with the 3D HD system (*P* < 0.003) with a median completion time of 134 s compared to 158 s with the 2D 4K system. The experts were also faster with the 3D HD system, but this was not significant (*P* < 0.460) (Tables 1, 2, and 3).

### Task 3: hot wire

The error ratio in the expert and student groups was significantly lower when using the 3D 4K system (*P* < 0.001 and *P* < 0.001, respectively). The students made nearly twice as many mistakes as the experts with the 3D HD system (median 17 vs. 8) and the 2D 4K system (median 57.5 vs. 32). Both groups were faster in completing the task using the 3D HD system. However, only the students were significantly faster (*P* < 0.003) (Tables 1, 2, and 3).

### Task 4: threading

Both the students and experts showed a significant reduction in the time spent completing this task (*P* = 0.001 and *P* < 0.006). The students and experts performed the task almost twice as quickly using the 3D HD system compared to the 2D 4K system. In addition, the experts were more than twice as fast as the students independent of the system used.

Both experience levels showed a decrease in the number of mistakes made when completing this task using the 3D HD system; however, students and experts had no significant reductions in the number of mistakes made between the two image systems (*P* < 0.059 and *P* < 0.275). (Tables 1, 2, and 3).

### Questionnaire results

**Fig. 3:**
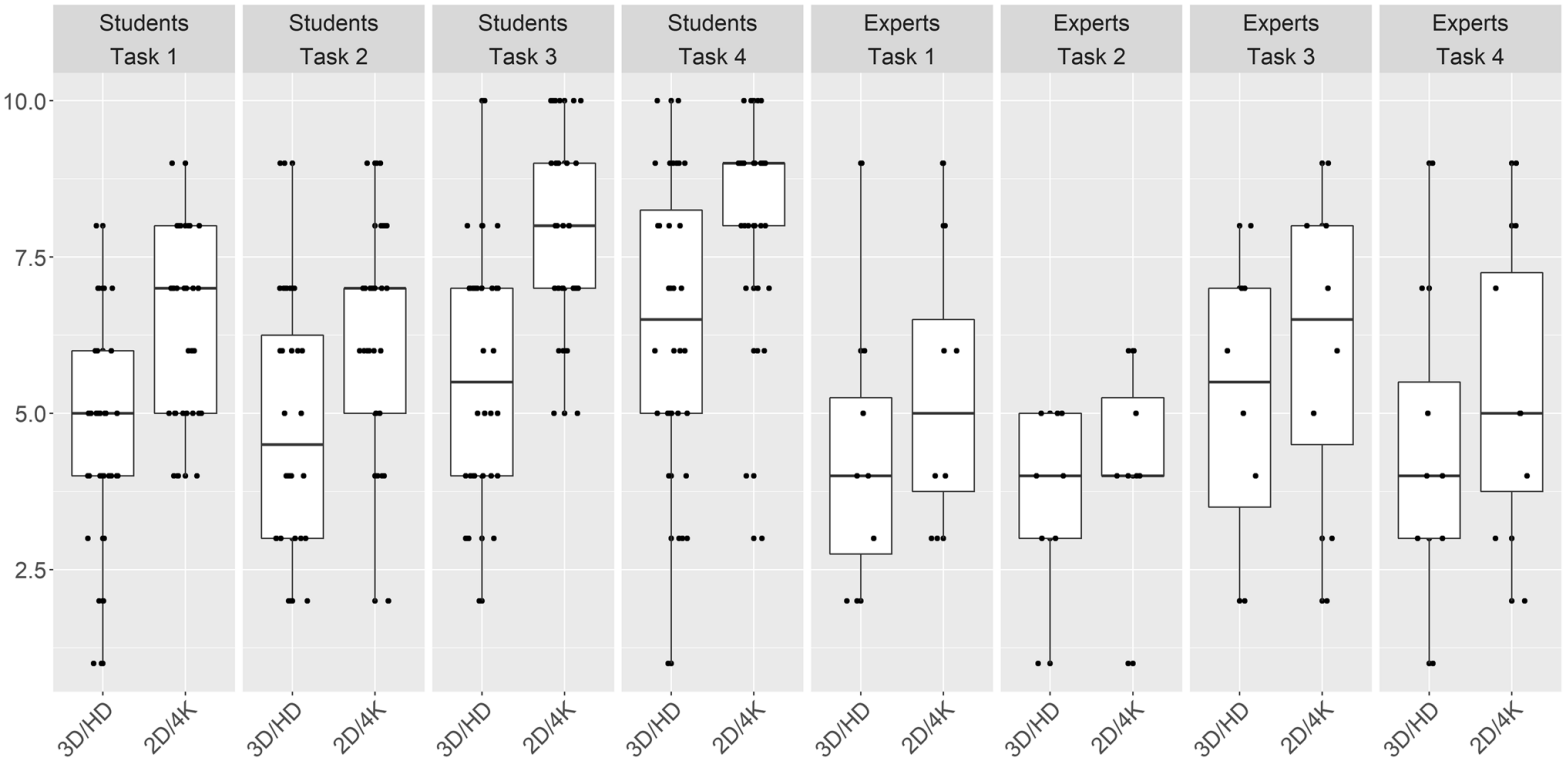
The subjective rating of both experience groups on how challenging they found each task when using the two different image system. 1 = not challenging, 10 = extremely challenging

**Fig. 4:**
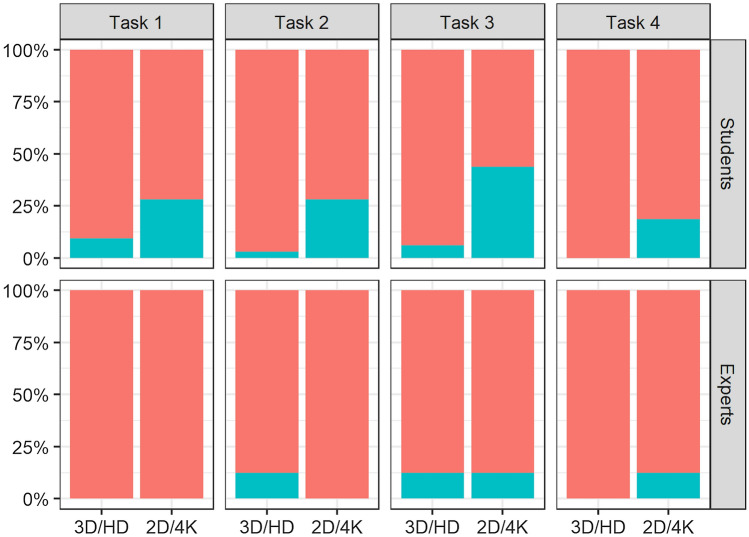
The percentage of students and experts who reported being acclimated to the image system for each task are represented in red

All of the study participants rated the tasks as more challenging when using the 2D 4K system (Fig. 3). Overall, the participants acclimated themselves to both of the imaging systems; although, adapting to the 3D HD system appeared to be easier, especially for the students (Fig. 4).

**Fig. 5:**
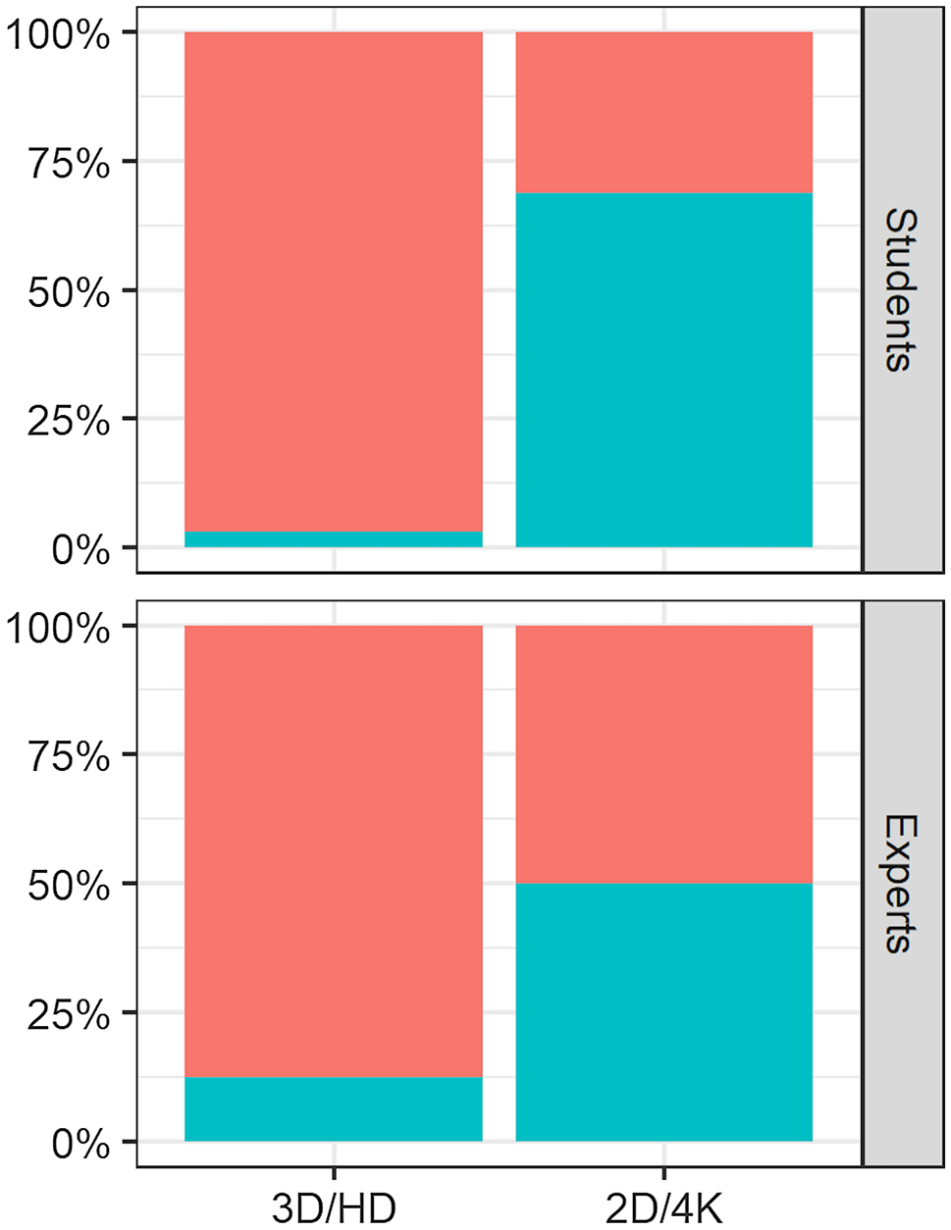
The percentage of students and experts who found the imaging systems intuitive are represented in red

However, 35% of the participants reported that the goggles used during the tasks were distracting, and one participant experienced nausea while using the 3D system. Still, more than 96% of the students and 87.5% of the experts considered the 3D HD system to be intuitive compared to 31.25% and 50%, respectively, for the 2D 4K system (Fig. 5).

**Fig. 6:**
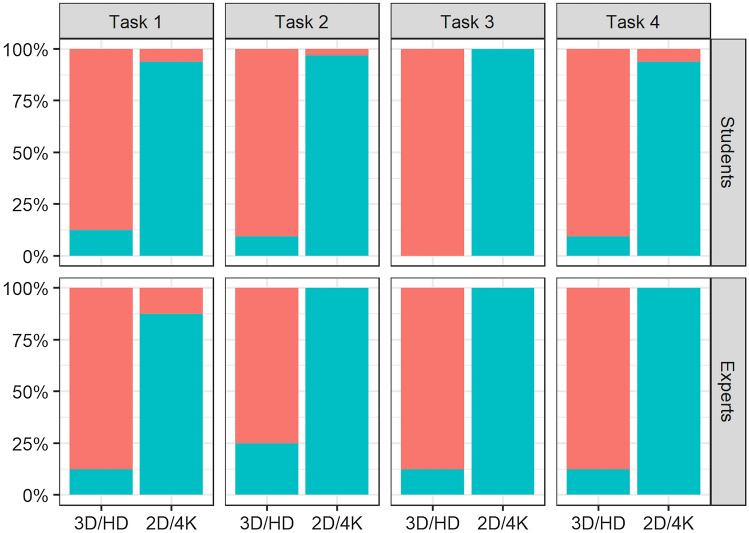
The percentage of students and experts who found the imaging systems were advantageous during the performance of the tasks are represented in red

More than 75% of participants in both experience groups found the 3D HD imaging system to be helpful in performing all four tasks versus 12.5% for the 2D 4K (Fig. 6).

No correlation was found between playing videogames or sports regularly and the imaging system performance of the participants. Age, sex, and specialization was also found to have no effect on the time needed to complete the tasks. However, the time needed to carry out the tasks was found to decrease with an increasing number of operations a participant performed per year. In contrast, the number of years performing laparoscopies had no effect.

At the student level, there was a correlation showing that the higher the subjects rated the challenge of the task, the more time they needed to complete it. For the experts, no correlation was observed. In addition, in both experience groups a decrease in concentration correlated with an increase in the time needed to complete the tasks.

## Discussion

The results of this study suggest the 3D HD system yields significantly better results in time and precision compared to a 2D 4K system, using a pelvitrainer model. The use of the 2D 4K imaging system showed no advantages in any of the performed tasks in comparison to the 3D HD imaging system which emphasizes the results of previous studies showing 3D HD as being partially superior to the 2D 4K system [23, 24]. The crossover study using the Williams design was chosen to prevent selection and learning effect biases.

The tasks were chosen to imitate the skills and scenarios found during laparoscopic operations. We selected abstract surgical tasks because more realistic ones, such as making knots or performing a myomectomy, would have given the expert group a greater advantage over the students. Though we did not prove the validity of each task in the operating theater, the tasks reached construct-validity within this and our previous study [14] as the method was capable of distinguishing the experienced surgeon from the inexperienced students. Additionally, face validity was achieved as some of the experts confirmed the value of the exercises in terms of realistic laparoscopic training [27]. In all of the tasks, ambidexterity was required.

Task 1 (mountain relief), combined depth perception, ambidexterity, and prevision. The 3D 4K system demonstrated its superiority in this task, with students being significantly faster and making fewer mistakes compared to their performance on the 2D 4K system. The experts reaffirmed these results by also requiring significantly less time and making fewer mistakes in the 3D HD system. In Task 2, the expert and student groups spent less time and made fewer mistakes when using the 3D HD system, but only the students were significantly faster with the 3D HD system. The improved performance in both groups may be due to the task requiring more grasping and targeting, with less need for depth perception.

However, in task 4, which also involved grasping and targeting, both experience groups displayed a significant decrease in the time spent completing the task while using the 3D HD system. For this task, the additional threading of the needle was likely facilitated by the better depth perception of the 3D HD system. Difficulty in threading the needle could also explain why the experts were more than twice as fast as the students, independent of the system that was used. The largest difference in mistakes made between the two imaging systems occurred during task 3. The experts were able to reduce their mistakes by more than 300%, and the students by 400%, when using the 3D HD system. The mistakes were strongly based on the difficult depth perception issue and were, therefore, simplified by the 3D HD system (Fig. 3). Overall, depth perception was essential for performing of all of the tasks, and this perception is likewise needed in laparoscopic surgeries involving patients [28]. Therefore, we believe that as the 3D HD system can facilitate laparoscopy in a pelvitrainer model, it will likely do so with patients as well.

The results of our questionnaire confirmed the superiority of the 3D HD system in terms of being more intuitive, providing better acclimation to the imaging system, ease of task performance, and finding the tasks less challenging. In addition, we looked for a correlation between participants’ answers and their performance results. We found a slight correlation between years of experience and task duration. However, there was no correlation between how much a student or expert played videogames or the type of sports they played.

There were some limitations of our study. The major limitation was that the participants performed simple tasks in a pelvitrainer model. More challenging tasks would have proven more accurate representation of the 3D HD and 2D 4K system. In addition, we only compared the 3D HD system with the 2D 4K imaging system, even though 2D HD is more common in operating rooms. However, our results remain useful as studies comparing 3D HD with 2D HD have been reported extensively, while only few exist for the 2D 4K system [23–25, 29]. Moreover, the future of laparoscopy probably will use the 4K or even the 8K system, thus further necessitating a comparison of the 3D and 2D imaging systems in 4K resolution. Another limitation concerns the small sample of experts. This is partly the result of the strict inclusion criteria for the experts and the difficulty in recruiting them. While experts had extensive experience with the 2D system, they achieved better, and often significantly better, results with the 3D HD system for all of the tasks. It is plausible that these numbers would remain significant if a larger sample size of experts were to be tested. However, other comparative studies exploring the role of 3D HD, 2D HD, and 2D 4K systems in laparoscopy have used similar numbers of subjects [22–24, 29]. Additionally, a general limitation of the 4K technology is the requirement of large size monitors or, if that is not given, close distance of the surgeon to the monitors to provide the full benefit of 4K. On the other hand, 3D technology is often still not available in the clinic despite promising study results and the recommendation of the European Association of Endoscopic Surgery (EAES) [17]. The results of our study emphasize the need to apply the 3D technology in the current laparoscopic practice. However, further randomized prospective studies performed in the operating room on human patients should be and are already undertaken to confirm the superiority of the 3D HD over the 2D 4K system in the clinical setting [30–32].

## Conclusion

The results of this study show that the addition of 4K does not alter box trainer performance in 2D compared to 3D HD systems. It could confirm that the 3D imaging system has advantages in terms of time and precision in comparison with the 2D system, and the resolution of 4K does not appear to provide the desired effect for 2D. Experts and beginners can benefit from the 3D HD system in achieving faster performances with fewer mistakes.

These results should be confirmed with prospective studies in surgical practice. We hope this study raises awareness regarding the advantages of applying the 3D HD imaging system since the use of it is still limited due to higher costs and unfamiliarity.

## Data Availability

The datasets analyzed for this study are available on https://datadryad.org/stash/share/wMK3x4mPekH6foD-bsY21KpX4ICyLwp7dIsv6KmOXpM.
